# Metastatic prostate cancer mimicking a rectal cancer: a case report

**DOI:** 10.3332/ecancer.2021.1295

**Published:** 2021-09-23

**Authors:** Alfredo V Chua, Alvin Christopher S Chu, Ashraf A Tawasil, Michael D San Juan

**Affiliations:** 1 Division of Medical Oncology, Department of Medicine, University of the Philippines - Philippine General Hospital, Taft Avenue, Ermita, Manila 1000, Philippines; 2 Department of Laboratories, University of the Philippines - Philippine General Hospital, Manila 1000, Philippines; 3 Division of Gastroenterology, Department of Medicine, University of the Philippines - Philippine General Hospital, Manila 1000, Philippines

**Keywords:** prostatic neoplasms, rectal neoplasms, case report

## Abstract

The most common presenting symptoms of prostate cancer, a common cancer in males worldwide, are lower urinary tract symptoms. In rare cases, however, urinary symptoms may not be apparent, and patients can present with gastrointestinal symptoms instead. Even rarer is the involvement of non-regional lymph nodes such as the cervical nodes. Here, we report a case of a 50-year-old male who initially presented with constipation and an enlarging left lateral neck mass. Further work-up revealed metastatic prostatic adenocarcinoma and the patient dramatically responded to chemotherapy, androgen deprivation therapy and bone support therapy. This case highlights the importance of considering a prostate malignancy in a male patient presenting with gastrointestinal symptoms and a neck mass even in the absence of lower urinary tract symptoms. Serum prostate specific antigen, pathologic findings and immunohistochemistry staining are important to guide the clinician in making the correct diagnosis and treatment.

## Introduction

Prostate cancer was the second most common cancer in men worldwide in 2020 [1]. An estimated 1.4 million new cases of prostate cancer were recorded in the same year, making up around 7.3% of all new cancer cases in both sexes and 14.1% in men [1]. In the Philippines, prostate cancer was the third most common cancer among males in the same year [2]. There were an estimated 8,242 new cases in the same year and the incidence rate continues to rise annually [2, 3].

Because of the anatomic location of the prostate gland, the most common presenting symptoms of prostate cancer are symptoms related to the urinary tract such as difficulty in urination, haematuria and pelvic pain [4]. Rarely, urinary symptoms may not be apparent [5], and patients can present with gastrointestinal symptoms like constipation as presented in this case. Patients may also present with rectal urgency, bowel obstruction and gastrointestinal bleeding [4]. In a series of men with prostate cancer invading the rectum, 51% presented with gastrointestinal symptoms prior to the establishment of the diagnosis [6]. Currently, the diagnosis of prostate cancer is suspected among asymptomatic males based on an abnormal serum prostate specific antigen (PSA) and digital rectal examination (DRE). However, Filipino patients are still diagnosed in the late stages when they already present with urinary symptoms [7].

More unusual is the presentation of prostate cancer as a left lateral neck mass as illustrated in this case. The most frequent sites of metastasis of prostate cancer are the axial skeleton due to the rich Batson venous plexus and the pelvic and retroperitoneal lymph nodes [8–10]. Involvement of non-regional lymph nodes such as the cervical nodes as the initial presentation is extremely rare with an estimated incidence rate of 0.4% or less [8, 10, 11]. The left supraclavicular fossa is the most common reported site of extra-skeletal non-regional lymphatic spread [12]. The regional lymph nodes such as the obturator, hypogastric and presacral nodes are usually involved first [8, 13]. However, further spread may occur via the iliac and paraaortic nodes to the cisterna chyli and thoracic duct where the cancer cells can gain access to the systemic blood circulation via the left subclavian vein and ultimately lodging in the left supraclavicular nodes in a retrograde fashion [8].

### Patient information and clinical findings

A 50-year-old male farmer, past heavy smoker (10 pack-years), with no known co-morbidities, presented with a 1-year history of gradually enlarging nontender left neck mass, initially approximately 2 × 2 centimetres in size. He also complained of constipation but no abdominal pain, decreased stool calibre, melena or haematochezia. There were no reported lower urinary tract symptoms such as dysuria, urinary frequency, nocturia, haematuria, straining, hesitancy, urgency, intermittent stream, dribbling or feeling of incomplete bladder emptying. There were also no symptoms pertaining to a thyroid disease. There was no family history of any malignancy.

The left lateral neck mass was progressively enlarging in size which prompted consult at a local hospital where a neck ultrasound was done showing multiple, coalescing complex foci in the left lateral neck area, the largest of which measures 2.8 cm with perilesional uptake on Doppler study. The thyroid gland was normal in size with homogenous parenchymal echogenicity. Thyroid function tests were also normal. A fine needle aspiration biopsy of the neck mass showed atypical epithelial cells.

No masses, nodules, haemorrhoids or tenderness were palpated on initial DRE. There was good sphincter tone. No blood was seen per examining finger.

### Diagnostic assessment

**Figure 1.**
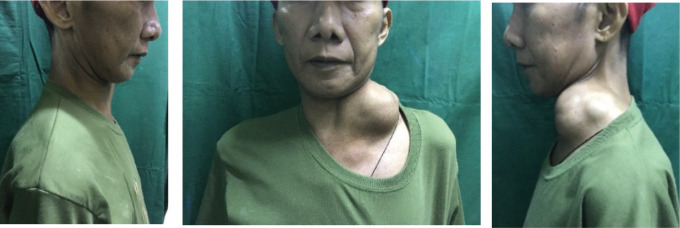
Left lateral neck mass of the patient measuring approximately 7 × 7.5 cm in size before initiation of treatment.

**Figure 2.**
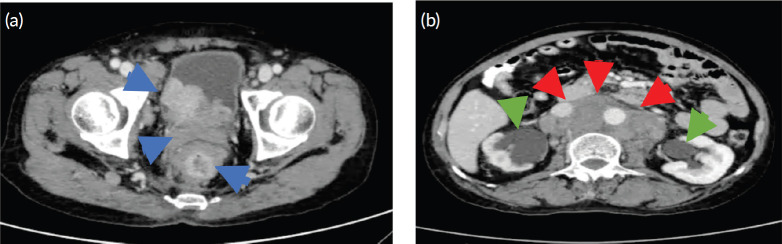
Representative axial cut images of the abdominopelvic CT scan with oral, rectal and intravenous contrast. (a):. The prostate gland is enlarged and converted to a heterogenous mass 6.8 × 5.2 × 5.4 cm in size and a volume of 99.3 cc with intravesical extension and cannot be separated from a rectal mass (blue arrows). (b): Confluent, matted, markedly enlarged retroperitoneal lymph nodes (red arrows) and dilated urinary collecting systems and ureters (green arrows).

**Figure 3.**
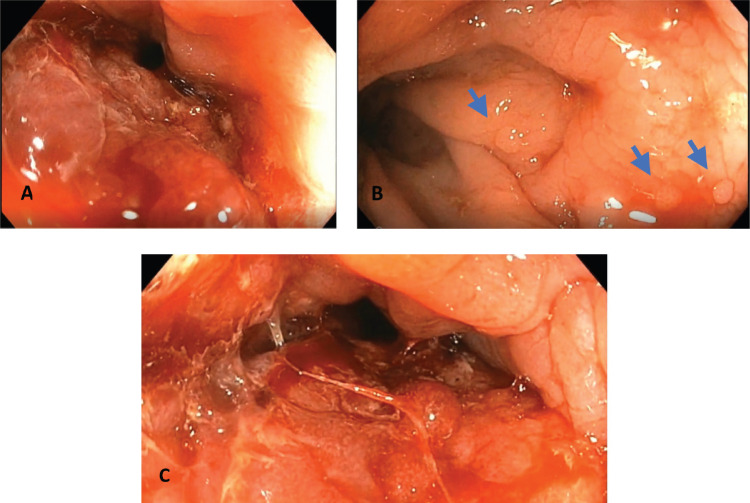
Representative images from the colonoscopy. (a): Circumferential rectal mass approximately 70% obstructing with noted inflamed nodular and friable mucosa. (b): Normal colonic mucosa beyond the mass with noted colonic polyps shown in blue arrows. (c): Circumferential rectal mass with noted scope dilated colonic lumen. Multiple biopsies were taken.

Weight loss, occasional hypogastric pain, persistent constipation and continuous enlargement of the neck mass to approximately 7 × 7.5 cm (Figure 1) prompted the patient to seek consult at our institution. Abdominopelvic computed tomography (CT) scan with oral, rectal and intravenous contrast (Figure 2) showed markedly enlarged lymph nodes in the retroperitoneum encasing the abdominal aorta and iliac vessels and pelvic sidewalls. Bilateral kidneys show dilated collecting systems and ureters. The prostate gland cannot be completely separated from a rectal mass. A sub-centimetre hypodense cystic nodule in segment IVA of the liver was seen which was considered benign. Multiple, variable-sized, mixed lytic and blastic lesions were noted throughout the thoracolumbar vertebrae and sacrum and all pelvic bones, left proximal femur and some of the included ribs, which were deemed metastatic. Carcinoembryonic antigen was 5.942 ng/mL. Chest X-ray showed a soft tissue density at the left neck region with intrathoracic extension displacing the trachea to the right. Colonoscopy was done which showed a circumferential mass spanning 10 to 20 cm from the anal verge. The mass partially obstructed 70% of the colonic lumen and was noted to have areas of nodular, friable and oedematous mucosa. Standard colonoscope was not able to pass through the narrowing. The mass was scope dilated upon switching to a paediatric colonoscope showing grossly normal appearing colonic mucosa beyond with multiple sub-centimetre Paris 1s NICE (Narrow-band Imaging International Colorectal Endoscopic Classification) rectal polyps ranging from approximately 0.1 to 0.2 cm in size located less than 5 cm beyond the mass. Multiple biopsies were taken along the circumferential mass. Figure 3 shows representative images from the colonoscopy.

**Figure 4.**
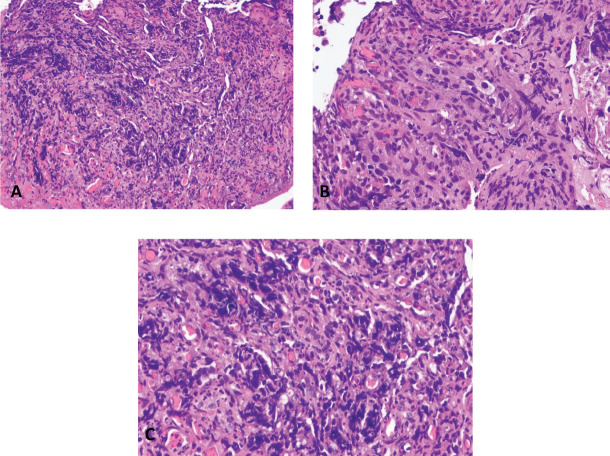
Rectal mass, biopsy. (a): Haematoxylin and eosin (H & E) stain, 200× magnification. (b and c): H & E stain, 400× magnification.

**Figure 5.**
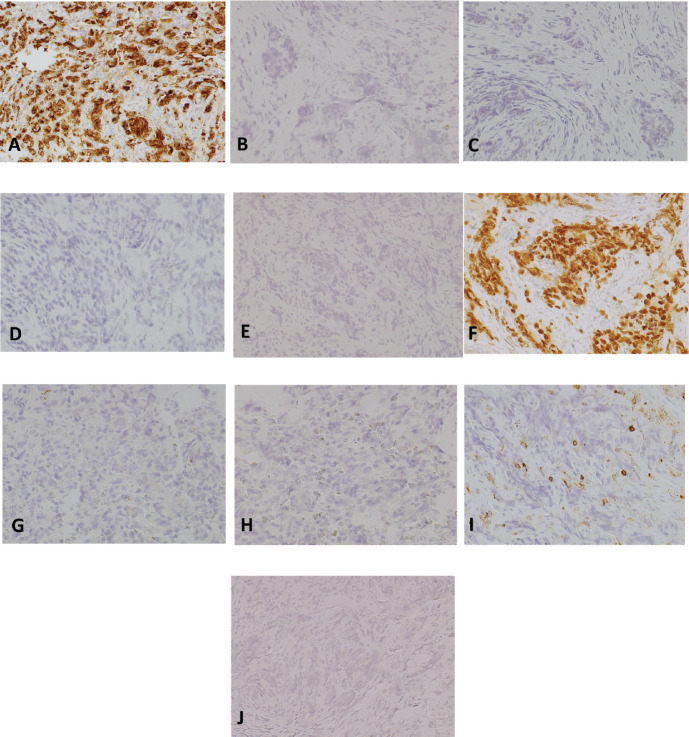
Immunohistochemistry studies, 200×magnification. (a): pancytokeratin/cytokeratin AE1/AE3. (b): CK7. (c): CK20. (d): CDX2. (e): p63. (f): NKX3.1. (g): synaptophysin. (h): chromogranin. (i): LCA. (j): TTF-1.

Biopsy of the rectal mass showed medium to large sized atypical cells with high nuclear to cytoplasmic ratio, arranged in poorly formed glands and as individual cells with enlarged, pleomorphic, hyperchromatic nuclei with prominent nucleoli (Figure 4). A histopathologic diagnosis of a poorly differentiated carcinoma was made with recommendations for immunohistochemistry studies for further evaluation. Immunohistochemistry studies showed that the tumour was positive for pancytokeratin and NKX3.1; and negative for CDX2, LCA, synaptophysin, chromogranin, CK7, CK20, TTF-1 and p63 (Figure 5). The histopathologic findings, together with the immunohistochemistry staining patterns favour the prostate gland as the primary site of malignancy. Immunohistochemistry staining for PSA was not done due to unavailability in our institution. Baseline serum PSA was >100.00 ng/mL.

### Therapeutic interventions

The patient was then treated as a case of metastatic prostate adenocarcinoma. He received six cycles of docetaxel at 75 mg/m^2^ every 3 weeks with prednisone 5 mg twice daily, androgen deprivation therapy with goserelin 10.8 mg subcutaneously every 3 months and bicalutamide 50 mg once daily, and bone support therapy with denosumab 120 mg subcutaneously every month.

### Follow-up and outcomes

**Figure 6.**
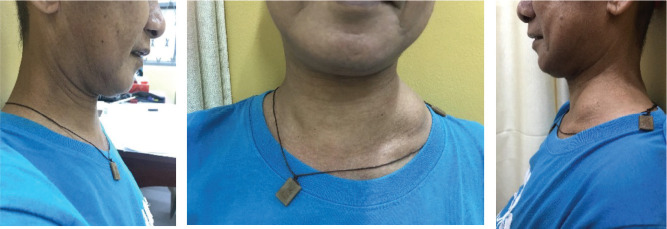
Left lateral neck mass of patient decreased to approximately 4 × 4 cm in size after 1 month of treatment (previously 7 × 7.5 cm).

**Figure 7.**
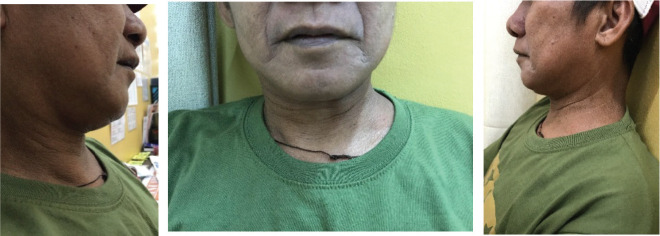
Left lateral neck mass not anymore evident by inspection and barely palpable at approximately 1.5 × 2 cm in size after 5 months of treatment.

After 1 month of treatment, there was drastic regression in the size of the neck mass to approximately 4 × 4 cm as shown in Figure 6. After 5 months of treatment, the neck mass was not anymore evident by inspection and was barely palpable at approximately 1.5 × 2 cm in size (Figure 7). The latest PSA taken after 5 months of treatment was 18.07 ng/mL. The patient is on regular follow-up in our institution and is continuing his androgen deprivation therapy and bone support therapy.

## Discussion

The incidence of advanced prostate cancer invading the rectum was estimated to be 1%–11% [5]. Despite their proximity, extension of a prostate malignancy to the rectum is rare because of the presence of the Denonvillier’s fascia, a thick capsule which separates the rectum from the prostate gland [4, 14]. Aside from direct extension through this fascia, a prostate malignancy can also invade the rectal wall by lymphatic metastasis or by seeding during a needle biopsy [4]. It is difficult to clinically distinguish a primary prostate malignancy invading the rectum from a primary rectal malignancy invading the prostate gland [14]. Furthermore, colorectal masses in patients with known prostate cancer may also be due to a second primary colorectal malignancy. Reports have also shown that primary colorectal tumours are nearly impossible to distinguish from metastatic secondary colorectal tumours [15]. This makes it exceedingly difficult to diagnose secondary colorectal tumours macroscopically on colonoscopy alone as was observed in this patient. Furthermore, the DRE findings can be normal in as much as 58% of patients with previously unrecognised metastatic prostate cancer presenting with left supraclavicular lymphadenectomy [9], as was seen in our patient.

The use of endoscopic ultrasonography such as in transrectal ultrasound or endoscopic ultrasound may be recommended to aid in the overall diagnosis of rectal masses. The advantages have been seen in its use in early primary rectal cancer where endoscopic ultrasound has been used in staging and fine-needle aspiration biopsy technique [16]. In prostate cancer patients, transrectal ultrasound has been documented to aid in screening along with PSA and DRE [17]. However, no clear-cut recommendations have been made to use endoscopic ultrasonography to differentiate the invasion of a prostate malignancy from a primary rectal cancer without histopathologic diagnosis.

Preoperative pelvic magnetic resonance imaging is recommended for select patients with rectal masses to define the extent of possible surgery, as it is the most accurate test to define locoregional clinical staging [16]. However, this was not done in our patient anymore because it was not readily available in our setting and the CT scan was deemed sufficient to adequately stage the patient and decide on the treatment plan.

The morphologic features of prostate and colorectal adenocarcinoma are also similar. Both malignancies may present as well differentiated tumours showing prominent gland formation with cells characteristic of their primary site of origin to poorly differentiated tumours with poorly formed glands and interspersed individual malignant cells. Both tumours may present with atypical cells showing cellular and nuclear pleomorphism and prominent nucleoli with only ‘soft signs’ pointing to either a colorectal (prominent necrosis, columnar cellular morphology) or a prostatic (microacinar architecture) primary [18–20]. These features are neither exclusive nor definitive for colorectal or prostatic adenocarcinoma, hence the importance of using immunohistochemistry to assist in the determination of the primary site of origin. A pathologic misdiagnosis can lead to inappropriate treatment plans and adverse outcomes [4]. Pancytokeratin or cytokeratin AE1/AE3 is an immunohistochemistry stain that detects most cytokeratin proving or ruling out the epithelial nature of a tumour [21]. In the case presented, the tumour cells showed diffuse, cytoplasmic staining. Specific cytokeratins like CK7 and CK20 are used in combination to help narrow down the differentials for carcinomas of unknown origin [21]. CK7 and CK20 were both negative. NKX3.1 is a protein expressed primarily in adult prostate and antibodies against it (which are used in immunohistochemistry studies). This has been shown to be a highly sensitive and specific marker for carcinomas of prostatic origin [22]. In this case, the tumour cells were diffusely positive. Even though immunohistochemistry staining for PSA was not done due to unavailability in our institution, NKX3.1 has comparable sensitivity and specificity with PSA in identifying metastatic prostate adenocarcinoma [22]. The immunohistochemistry staining pattern, together with the histology, pointed to a prostatic primary.

The clinical scenario presented in this case can lead to diagnostic pitfalls. Even though prostate cancer is a common malignancy in males, it is often overlooked as the primary site in patients presenting with cervical lymphadenopathy [9]. A prostate malignancy should be considered in a male patient presenting with a left cervical lymphadenopathy [10] and gastrointestinal symptoms even in the absence of lower urinary tract symptoms. Serum PSA should be included in the work-up [14] and pathologic and immunohistochemistry findings are important to guide the clinician in determining the definite diagnosis. The recommended chemotherapy, androgen deprivation therapy and bone support therapy are effective even in the advanced or metastatic setting. Prognosis can be relatively good with prolonged survival given the appropriate diagnosis and treatment [10].

## Conclusion

Even though prostate cancer is a common malignancy in males, it is often overlooked as the primary site in patients presenting with cervical lymphadenopathy. It is important to consider a prostate malignancy in a male patient presenting with gastrointestinal symptoms and a neck mass even in the absence of lower urinary tract symptoms. Serum PSA, pathologic findings and immunohistochemistry staining are important to guide the clinician in making the correct diagnosis and treatment.

## Patient perspective

‘We are greatly thankful to all my doctors. For more than a year, my neck mass was continuously enlarging in size. For a long time, I was being worked-up to search for the real site of my cancer. When my doctors were able to pinpoint where it really was and I was started on treatment, I noticed a drastic improvement in my condition. Aside from the mass decreasing in size to almost nothing, my appetite also improved, and I gained weight. Even though my medications are expensive for a farmer from the province like me, we are doing our best to follow my doctors’ instructions. Even if I have stage 4 cancer, there is still hope’ (Translated from Filipino).

## Conflicts of interest

The authors declare that they have no conflicts of interest.

## Declaration of sources of funding

No funding was received by the authors with the preparation of this manuscript.
